# Prevalence and predictors of having no general practitioner - analysis of the German health interview and examination survey for adults (DEGS1)

**DOI:** 10.1186/s12875-019-0976-x

**Published:** 2019-06-15

**Authors:** Judith Tillmann, Marie-Therese Puth, Klaus Weckbecker, Manuela Klaschik, Eva Münster

**Affiliations:** 1 Institute of General Practice and Family Medicine, University of Bonn, Sigmund-Freud-Str. 25, 53127 Bonn, Germany; 2 Department of Medical Biometry, Informatics and Epidemiology (IMBIE), University Hospital of Bonn, Sigmund-Freud-Str. 25, 53127 Bonn, Germany

**Keywords:** General practice utilization, GP, Healthcare, DEGS, Medical care, Family doctor, Public health

## Abstract

**Background:**

Although patients in Germany are generally free to choose their primary healthcare provider, this role should mainly be assumed by general practitioners (GPs). While some predictors of the frequency of use of GP services have been reported in international studies, there is still a lack in knowledge what could deter people from contacting a GP in Germany. To improve healthcare, it is important to identify characteristics of people without a GP.

**Methods:**

This cross-sectional analysis was based on the first wave of the “German Health Interview and Examination Survey for Adults” (DEGS1) conducted by the Robert Koch Institute in 2008–2011. Descriptive analyses and multiple logistic regression by gender were performed to analyze the association between having no GP and age, gender, residential area, socioeconomic status (SES), marital status, working hours per week, general state of health, chronic diseases and health insurance.

**Results:**

Overall, 9.5% (95% confidence interval (CI): 8.4–10.7) of the 7755 participants stated to have no GP, more often men (11.4%) than women (7.6%). Life in urban areas (big cities vs. rural: adjusted odds ratio (aOR): 2.9, 95% CI: 2.1–3.9), younger age (18–29 years vs. 65–79 years: aOR: 4.4, 95% CI: 2.5–7.7) and the presence of chronic diseases (yes vs. no: aOR: 0.4, 95% CI: 0.3–0.6) showed significant associations of not having a GP. For men, the type of health insurance (private vs. statutory: aOR: 2.1, 95% CI: 1.5–3.0; other vs. statutory: aOR: 2.1, 95% CI: 1.4–3.1) and for women, SES (low vs. medium: aOR: 1.8, 95% CI: 1.2–2.7; high vs. medium: aOR: 2.1, 95% CI: 1.4–3.0) increased the risk of having no GP.

**Conclusions:**

Our analysis offers new insights into the use of GPs in Germany and revealed differences between men and women. Public health strategies regarding access to a GP have to focus on men and on women with a low SES. Further analyses are needed to determine whether men with private health insurance prefer to consult a specialist rather than a GP. For young adults, improving the transition process from a pediatrician to a GP could fill a gap in health care.

**Electronic supplementary material:**

The online version of this article (10.1186/s12875-019-0976-x) contains supplementary material, which is available to authorized users.

## Background

The frequency and predictors of the use of general practice services have rarely been addressed in German research. One of the most important components of the German health care system is characterized by the free choice of a healthcare provider (§ 76 Code of Social Law Volume V) for each of the 82.4 million citizens [1]. It is intended that the GP is the first point of contact for any health problem and acts as a guide at all steps of treatment [2]. GPs are best placed to assess what therapy is necessary or helpful for their patient. Better health outcomes through GP-centered healthcare, especially among older or chronically ill patients, have already been reported [3, 4]. The role of general practitioners (GPs) in Germany is therefore of major importance in the health care system. Although it is advocated that every German citizen should have a GP in case of any possible health problem, research has been limited to the frequency of use of GPs. However, it is important to start research earlier to find out what drives or discourages people from contacting a GP. Thus, knowledge of the effect of various sociodemographic and health characteristics completes the overall picture that is necessary to develop more effective health measures in order to raise awareness of the importance of a GP.

Every employed citizen in Germany is obliged to be insured by a statutory health insurance up to an income of 4350 euros per month and family members who do not earn a living are insured free of charge. Citizens subject to social welfare programs are also covered by statutory health insurance [5]. In total, 87.7% of the German population is covered by statutory health insurance [5]. Citizens with a higher income as well as the self-employed and civil servants have the option of a private health insurance (11.5% of the population) [5]. According to the European Social Survey, a low socioeconomic status (SES) is associated with an increased use of general practice services [6]. In Danish studies, unemployment and a low educational level increased the use of GPs most [7, 8]. According to the “Quality and Costs of Primary Care in Europe” (QUALICOPC) study, financial factors were the main predictors of access to primary health care [9]. In contrast, Hessel et al. reported only a small influence of socioeconomic factors on the number of contacts with GPs among people aged 60 years and over in Germany [10]. Both, in a Danish cohort study (OR: 1.95; 95% CI: 1.85–2.06, aOR: 1.26; 95% CI: 1.09–1.47) [8, 11] and the “German National Health Interview and Examination Survey 1998” (GNHIES98) [8, 11], women were associated with a more frequent use of GPs. In addition, Danish studies showed a clear gender difference in the number of consultations (4.1 per year among women vs. 2.8 among men) [7]. The importance of the residential area remains contradictory: some studies show that people in urban areas use medical care more often than the rural population [11, 12] while others found that there is no impact of the residential area [7]. In a former analysis of a German study, it was also reported that unmarried or married people visited their GPs more often than divorced or widowed ones [7].

Pain medication, a poor individual health status and having one or more health problems were identified as important factors that increased the use of GP services in Australian studies [12]. Jørgensen et al. illustrated that hypertension (OR: 1.63; 95% CI: 1.59–1.67), mental illness (OR: 1.63; 95% CI: 1.61–1.66), diabetes (OR: 1.56; 95% CI: 1.47–1.65) and angina pectoris (OR: 1.28; 95% CI: 1.21–1.34) were associated with the use of GP services [7]. However, comparability of these studies is limited due to methodological differences in health care systems and other characteristics such as age and gender.

The aim of this study was to examine the relationship between a number of sociodemographic and health characteristics and having no GP in Germany.

## Methods

This cross-sectional analysis was based on the public use file (PUF) of the “German Health Interview and Examination Survey for Adults” (DEGS) conducted by the Robert Koch Institute [13]. The Robert Koch Institute is a federal institution financed by the German Federal Ministry of Health and is responsible for the research of infectious diseases and, within the framework of health monitoring, for the analysis of national long-term public health trends [14]. The PUF contained interview and examination data from the first wave of the survey (DEGS1) which was conducted between November 2008 and November 2011 with more than 8000 adults. DEGS1 consisted of interviews, self-administered questionnaires, standardized tests and measurements to provide information on various self-reported health conditions, current medications as well as sociodemographic characteristics [15–17]. The target population included people aged 18 to 79 years who lived permanently in Germany. Based on a two-stage stratified cluster sampling procedure, 180 sample points were determined based on a list of nationwide municipalities. Within the sample points, individuals were randomly selected from local population registries two months before the planned study period [15]. Eligible individuals were invited to participate in the survey by letter sent about five weeks prior to the survey visit [15]. DEGS1 included new randomly collected participants (response rate 42%) and former participants (response rate 62%) of the cross-sectional GNHIES98 study, also conducted by the Robert Koch Institute from 1997 to 1999 [18]. The cross-sectional analyses with the PUF were limited to 7987 participants aged between 18 and 79 years. Further details on the methodological procedures have already been published [15, 18].

The survey collected data on the utilization of different health care services, including information on the use of a GP. Whether or not participants have a GP was used as an outcome measure in the present analysis and was inquired by means of the following question: “Do you have a GP who is usually your first point of contact in case of any health impairment?”. If the question was affirmed, it was assumed that the participants had a GP. On the other hand, if the response was negative, it was concluded that these participants did not have a GP.

Potential factors associated with having no GP included in the analysis were age, which was categorized into four different groups: 18–29 years, 30–44 years, 45–64 years and 65–79 years. Four categories were distinguished regarding the residential area depending on the number of inhabitants within a community: rural area (< 5000 inhabitants), small town (5000 - < 20,000 inhabitants), medium-sized town (20,000 - < 100,000 inhabitants) and big city (100,000+ inhabitants). SES was based on a multidimensional index that included information on education, occupation and net household income of the participants. Each of the three dimensions was evaluated on a point scale from 1 to 7, resulting in a range of values from 3 to 21 for the combined index. Based on the distribution of the multidimensional index, it was divided into five equally sized groups (quintiles), which were used to classify low (1st quintile), medium (2nd to 4th quintiles) and high (5th quintile). Further details, such as the classification of the three dimensions, have already been published under [19]. The variable representing marital status was summarized into married (living together or apart), single and divorced/widowed. The availability of information on the usual number of working hours per week was limited to currently employed participants that were younger than 65 years. It was used to generate a variable (long working hours) with a cut-point at 50 h per week. The general state of health was dichotomized into the categories very good/good and average/poor/very poor. The data on the presence of chronic diseases (yes/no) was based on self-reported information for each participant. Health insurance was categorized into statutory health insurance, private health insurance and other (including no insurance, direct payer, foreign health insurance or any other kind of reimbursement).

Statistical analyses included absolute frequencies, percentages and 95% confidence intervals (CI). Differences between adults with and without a GP were examined using Chi-square tests for all categorical variables and a *p*-value < 0.05 was considered significant. Multiple logistic regression analyses with “having no GP” as dependent variable were performed for the total study population, and separately by gender. Adjustments for age, residential area, SES, marital status, long working hours per week, general state of health, chronic diseases and health insurance were added and adjusted odds ratios (aOR) with 95%-CI were determined. For all covariates, the amount of missing responses did not exceed 5%, so missing responses were allocated to the reference category in the regression analysis. All analyses were weighted according to the standardized weighting factor based on age, gender, federal region of residence, level of education, community class and nationality provided by the Robert Koch Institute in order to correct for any deviations of the DEGS1 study population from the German general population (reference date: 31th December 2010) [18]. For former participants of the GNHIES98, the re-participation rate was also considered within the weighting procedure. IBM SPSS Statistics (version 24) with the complex sample module was used [20].

## Results

The total number of participants was 7987. Of those, 232 participants were excluded from the analysis due to missing responses regarding the information on having a GP. Hence, the study population included 7755 participants of which 614 (9.5%) indicated that they did not have a GP (Table 1).

**Table 1:** Characteristics of the study population by having no General Practitioner (DEGS1)

	Study population	%^a^ with no GP	*p* value^b^
	n (%^a^)	(95% CI)	
Total	7755 (100)	9.5 (8.4–10.7)	
Gender			< 0.001
Male	3682 (49.7)	11.4 (10.0–13.0)	
Female	4073 (50.3)	7.6 (6.4–9.0)	
Age groups (years)			< 0.001
18–29	1063 (19.1)	17.9 (14.8–21.4)	
30–44	1693 (25.4)	11.8 (9.9–14.1)	
45–64	3051 (36.5)	6.6 (5.5–8.0)	
65–79	1948 (19.0)	3.3 (2.4–4.6)	
Residential area (inhabitants)			< 0.001
Big-city (100,000+)	2179 (31.0)	14.6 (12.3–17.3)	
Medium-sized town (20,000- < 100,000)	2244 (29.5)	8.0 (6.6–9.7)	
Small-town (5000 - < 20,000)	1904 (23.3)	7.3 (5.7–9.2)	
Rural (< 5000)	1428 (16.2)	5.5 (4.2–7.1)	
Marital status			< 0.001
Single	1670 (26.5)	15.9 (13.5–18.6)	
Divorced/widowed	957 (11.2)	6.2 (4.4–8.6)	
Married	5051 (62.3)	7.4 (6.3–8.6)	
Socioeconomic status			< 0.001
Low	1167 (18.9)	10.1 (7.9–12.7)	
Medium	4654 (60.6)	7.9 (6.7–9.2)	
High	1903 (20.4)	13.8 (11.4–16.5)	
Long working hours (≥50 h/week)			< 0.001
Long working hours	592 (8.3)	13.7 (10.8–17.3)	
Non-working/65+ years	3196 (36.9)	6.9 (5.7–8.4)	
No long working hours	3839 (54.9)	10.6 (9.1–12.3)	
General state of health			< 0.001
Very good/good	5723 (75.2)	10.9 (9.6–12.4)	
Average/poor/very poor	2005 (24.8)	5.1 (3.8–6.7)	
Chronic diseases			< 0.001
Any chronic disease	2504 (30.4)	3.7 (2.8–5.0)	
No chronic disease	4875 (69.6)	11.9 (10.4–13.6)	
Health insurance			< 0.001
Private	527 (6.7)	19.6 (15.5–24.5)	
Others^c^	468 (5.4)	16.0 (11.9–21.2)	
Statutory	6749 (87.9)	8.3 (7.2–9.6)	

^a^ Weighted results to match the German population structure on 31th December 2010

^b^ *P* values: Comparison between adults having a GP and having no GP

^c^ “Others” include no insurance at all, direct payer, a foreign health insurance or any other kind of reimbursement

Unweighted n may not add up to total n due to missing responses

Characteristics of the study population are summarized in Table 1: Having no GP was more prevalent among men (11.4%) than among women (7.6%). Regarding the effect of age, participants aged 18–29 years showed the highest rate to have no GP (17.9%). Participants from urban areas reported more frequently that they had no GP (14.6%) than participants living in rural areas (5.5%). For single participants (15.9%), for people with a low (10.1%) or high (13.8%) SES, and for participants who worked long (13.7%), it was more likely to have no GP. Participants with an average, poor or very poor general state of health as well as participants with chronic diseases stated more often to have a GP. In addition, people with a private (19.6%) or any other type of health insurance (16.0%) were more likely to be without a GP than people with a statutory health insurance (8.3%).

Gender, age, residential area, SES, the presence of chronic diseases and the type of health insurance showed significant associations with the odds of having no GP in multiple logistic regression analysis (Table 2). Not having a GP was more likely in young adults than in men and women in the oldest age group. Adults living in big cities had odds of not having a GP nearly three times higher (men: aOR: 2.7, 95% CI: 1.8–4.2; women: aOR: 3.0, 95% CI: 1.9–4.8) than men and women living in rural areas (Table 2). The presence of chronic diseases for men and women reduced the odds of having no GP (men: aOR: 0.4, 95% CI: 0.3–0.7; women: aOR: 0.5, 95% CI: 0.3–0.8) in comparison to adults without any chronic disease (Table 2).

**Table 2:** Predictors of having no General Practitioner: Adjusted odds ratios (aOR) with 95% confidence intervals (DEGS1)

	Total	Male	Female
	aOR^a^ (95% CI)	aOR^b^ (95% CI)	aOR^c^ (95% CI)
Gender			
Male	1.4 (1.2–1.8)	–	–
Female	ref.	–	–
Age group (years)			
18–29	4.4 (2.5–7.7)	3.4 (1.5–7.6)	5.8 (2.8–12.1)
30–44	3.0 (1.8–4.9)	2.6 (1.3–5.4)	3.2 (1.7–6.1)
45–64	1.9 (1.2–2.9)	1.8 (0.9–3.5)	1.9 (1.0–3.3)
65–79	ref.	ref.	ref.
Residential area (inhabitants)			
Big-city (100,000+)	2.9 (2.1–3.9)	2.7 (1.8–4.2)	3.0 (1.9–4.8)
Medium-sized (20,000- < 100,000)	1.4 (1.0–2.0)	1.2 (0.7–2.0)	1.7 (1.1–2.7)
Small-town (5000 - < 20,000)	1.4 (1.0–2.0)	1.3 (0.8–2.1)	1.4 (0.8–2.5)
Rural (< 5000)	ref.	ref.	ref.
Marital status			
Single	1.2 (0.9–1.7)	1.4 (0.9–2.3)	1.0 (0.6–1.6)
Divorced/widowed	1.1 (0.7–1.6)	1.4 (0.8–2.6)	0.8 (0.5–1.4)
Married	ref.	ref.	ref.
Socioeconomic status			
Low	1.5 (1.1–2.0)	1.3 (0.9–1.9)	1.8 (1.2–2.7)
Medium	ref.	ref.	ref.
High	1.4 (1.1–1.9)	1.1 (0.8–1.6)	2.1 (1.4–3.0)
Long working hours (≥50 h/week)			
Long working hours	1.2 (0.9–1.6)	1.1 (0.8–1.6)	1.5 (0.7–3.3)
Non-working/65+ years	1.1 (0.9–1.5)	1.1 (0.7–1.7)	1.2 (0.8–1.8)
No long working hours	ref.	ref.	ref.
General state of health			
Very good/good	1.2 (0.8–1.7)	1.4 (0.8–2.4)	1.0 (0.6–1.8)
Average/poor/very poor	ref.	ref.	ref.
Chronic disease			
Any chronic disease	0.4 (0.3–0.6)	0.4 (0.3–0.7)	0.5 (0.3–0.8)
No chronic disease	ref.	ref.	ref.
Health insurance			
Private	2.1 (1.5–3.0)	2.3 (1.6–3.4)	1.7 (0.9–3.2)
Others ^d^	2.1 (1.4–3.1)	2.4 (1.5–3.8)	1.6 (0.9–2.9)
Statutory	ref.	ref.	ref.

^a^ Adjusted odds ratios estimated from logistic regression for the total study population. Nagelkerke’s R^2^ = 0.14, 91% correctly classified

^b^ Adjusted odds ratios estimated from logistic regression restricted to male participants. Nagelkerke’s R^2^ = 0.13, 89% correctly classified

^c^ Adjusted odds ratios estimated from logistic regression restricted to female participants. Nagelkerke’s R^2^ = 0.14, 92% correctly classified

^d^ “Others” include no insurance at all, direct payer, a foreign health insurance or any other kind of reimbursement

Logistic regression analyses stratified by gender showed that, for both men and women, age, residential area and the presence of chronic diseases were associated with not having a GP. Differences between men and women were found in the type of health insurance and SES. For men, the type of health insurance was associated with having no GP (Table 2): Male participants with private (aOR: 2.3, 95% CI: 1.6–3.4) or any other health insurance (aOR: 2.4, 95% CI: 1.5–3.8) were more than twice as likely at risk as men with statutory health insurance. By contrast for women, SES showed a significant effect on the odds of having no GP (Table 2). In particular, female adults with low (aOR: 1.8, 95% CI: 1.2–2.7) or high SES (aOR: 2.1, 95% CI: 1.4–3.0) had higher odds of having no GP than female adults with medium SES. Additional analyses restricted to participants with valid data on all independent variables in regression (complete cases) showed similar results to the main analysis (see Additional file 1: Table S1).

## Discussion

Using data of 7755 adults aged between 18 and 79 years in Germany, the overall prevalence of having no GP was estimated to be 9.5% (men: 11.4%, women: 7.6%). Multiple logistic regression analyses showed that the odds of having no GP significantly decreased with age and the presence of chronic diseases. Odds were higher for adults living in urban areas. For males, the type of health insurance showed a significant association with having no GP: Men with a private or other type of health insurance more often had no GP than men with statutory insurance. By contrast for women, SES was significantly related with having no GP: Females with a high or low SES stated more frequently not to have a GP than females with a medium SES.

The comparison of our results with the existing literature is difficult, since in most studies the frequency of use of GP services instead of having a GP was the main focus of research. Yet, our finding that older and chronically ill participants were more likely to have a GP indicates that those are more often in need of a GP than young and healthier adults, as described in previous literature [7, 12]. Young participants suffer less often from health problems and chronic diseases and are usually not in need to have a GP or another specialized physician [21]. Moreover, older adults may be more familiar with the German health care system and they are used to have a GP as regular point of contact in case of any medical problem. They may also be more often in need to have a GP for e.g. regular health checks due to chronic diseases. On the other hand, participants in early adulthood may not have a GP as result of an insufficient transition process from a pediatrician to a GP. Gender differences in the presence of having a GP were in line with earlier findings and may be explained by a higher health awareness among women [22, 23]. In contrast to findings of the “German Health Update” (GEDA2012), this gender difference cannot be explained by a higher use rate of gynecologists among younger women [23]. Participants living in rural areas stated more frequently to have a GP than participants living in urban areas. One possible explanation might be that medical specialists are rare in rural areas in Germany and people therefore have no choice but to visit a GP [24–26]. On the other hand, people living in big cities may prefer to visit a specialist instead of a GP. A medically unjustified preference of patients to visit specialists instead of GPs would be problematic, as it could lead to misallocations. Such a preference would also lead to longer waiting times at specialists. In addition, this trend could mask a shortage of GPs and hamper the adaptation of medical care to the needs of the population. Further research into the reasons for the lower rate of having a GP in urban areas is needed to determine whether there is a real preference for specialists or whether the use of health care services is generally lower. Another explanation might be the higher work-related fluctuation of city dwellers, which may result in a frequent change of the GP. Although one would expect participants working 50 h or more per week to have less time to get in contact with a GP during regular consultation hours, an effect of the variable was observed in the bivariate analysis. That result may also be related to the often lower health awareness and poorer mental health of this population group, according to present literature [27, 28], and needs further research. Participants aged 65+ or those not in employment were more likely to have a GP, which may be due to a higher age of these participants. Many of them are already retired and may suffer from chronic or age-related diseases. In the present analysis these aspects have been found to be associated with a lower risk of having no GP. In contrast to results reported in most of the literature, not only participants with a low SES but also those with a high SES showed higher odds of having no GP [6–9]. Participants with a medium SES which accounts for about 60% of the DEGS1 study population were most likely to have a GP. Knowledge not only on diseases and symptoms but also on the German health care system may vary in SES groups. Participants of high SES, on the one hand, may prefer to consult medical specialists and thereby avoiding the primary care sector more often than those of medium SES. For adults with a low SES, on the other hand, the obligation to pay a “practice fee” of ten euros could have prevented them from contacting any physician. In Germany, the “practice fee” (in force from 2004 to 2012) was an additional fee payable once a quarter by every adult with a statutory health insurance when visiting a physician and was still in existence at the time of data collection. People with low SES may often not have been in contact with a physician for financial reasons. Especially the fact that SES appears to be a more important factor among female participants indicates the need for further research. A new aspect revealed by the analyses is that every fifth privately insured adult has no GP compared to every twelfth person with statutory health insurance. This difference was more pronounced among men. In Germany, a letter of referral from a GP is not mandatory, so people are free to arrange an appointment with a specialist themselves. Further, waiting times for an appointment with a specialist are significantly shorter for privately insured patients than for statutorily insured patients [29]. Accordingly, in a previous analysis of DEGS1 [21], privately insured adults consulted specialists (especially gynecologists, dermatologists, dentists) more frequently than GPs.

When considering our results, it should be noted that about 90% of the German population do have a GP. But the number of patients going to emergency rooms is growing steadily in Germany [30]. According to the recently published PiNo study, people visiting emergency departments are younger (42 years old on average), rather male (53%) and single (46%) [30]. Our results suggest that these people may be less likely to have a GP. In addition, more than half of the 1175 patients who visited emergency departments in the PiNo study rated their subjectively perceived treatment urgency as low, and about 35% of them had medical complaints for three days or longer [30]. Instead of going to an emergency department, these patients in particular could have consulted a GP. Hence it is possible that a misallocation of patients is not only due to the fact that the use of specialists is preferred, but also that more emergency departments are used instead of a GP. Further research is therefore necessary to determine whether adults without a GP have a higher number of contacts to emergency departments.

### Study limitations

DEGS1 provides a representative sample of the German population aged 18 to 79 years and for the first time, it enables an analysis of prevalence and predictors of not having a GP. Weighted results improve nationally valid conclusions. Still, it is possible that the results are biased, as all the information on the different characteristics is based on self-reported data. As in many other population-based surveys, chronically ill people may be underrepresented due to a potentially lower participation rate of sick people [17]. In addition, the presence of a GP does not mean that a participant actually uses the services of a GP. It only means that they know a doctor to whom they can turn first in the event of a medical problem. GPs as gatekeepers are extremely important in view of the highly relevant problem of over- and underdiagnoses in healthcare. We cannot rule out that participants who have recently moved had difficulties in answering the question of interest. It is possible that these participants used to have a GP in their previous residential area, but not in the new one. Thus, prevalence rates by residential areas may be distorted. Further research is necessary which also takes into account how long participants have been living in their area. Although it is likely that the participants’ migration background may have an impact on having no GP, it could not be assessed in this study due to lack of data in the PUF. Research that took the migration background of participants into account was for example considered in [31].

## Conclusions

Using a nationally representative sample, DEGS1 offers valuable results and new insights into the prevalence and the effect of different sociodemographic and health characteristics on the presence of having no GP in Germany. Almost every tenth person in Germany has no GP with differences between men and women. Public health strategies especially have to focus in particular on men, and women with a low SES. For men with private health insurance and women with high SES, further analyses are needed to determine whether they prefer to visit a specialist rather than a GP. Improving the transition process from a pediatrician to a GP could fill a gap in health care for young adults.

## Additional file

Additional file 1: **Table S1**. Predictors of having no General Practitioner: Adjusted odds ratios (aOR) with 95% confidence intervals (DEGS1) based on complete data (*n*=7.176). (DOCX 24 kb)

## Data Availability

The dataset analyzed during the present study is available from the Robert Koch Institute for researchers who meet the criteria for access [13, 32].

## Abbreviations

aOR: Adjusted odds ratio
CI: Confidence interval
DEGS1: German Health Interview and Examination Survey for Adults (first wave)
GNHIES98: German National Health Interview and Examination Survey 1998
GP: General practitioner
OR: Odds ratio
PUF: Public Use File
SES: Socioeconomic status
