# Supplementary material for: Prevalence and predictors of having no general practitioner - analysis of the German health interview and examination survey for adults (DEGS1)
Source: BMC Fam Pract. 2019 Jun 15;20:84. doi: 10.1186/s12875-019-0976-x (PMC6570899; doi:10.1186/s12875-019-0976-x)
Supplement: Supplementary file 1 — Table S1. Predictors of having no General Practitioner: Adjusted odds ratios (aOR) with 95% confidence intervals (DEGS1) based on complete data (n=7.176). (DOCX 24 kb) [file 12875_2019_976_MOESM1_ESM.docx]

**Additional file 1**

Additional analyses restricted to participants with valid data on all independent variables in regression (complete cases) showed similar results to the main analysis (table S1).

Table S1 Predictors of having no General Practitioner: Adjusted odds ratios (aOR) with 95% confidence intervals (DEGS1) based on complete data (n=7.176)

|  | **Total** | **Male** | **Female** |
| --- | --- | --- | --- |
|  | **aOR^a^ (95% CI)** | **aOR^b^ (95% CI)** | **aOR^c^ (95% CI)** |
| **Gender** | | | |
| Male | 1.4 (1.2-1.8) | - | - |
| Female | ref. | - | - |
| **Age group (years)** | | | |
| 18-29 | 4.2 (2.3-7.4) | 3.1 (1.4-6.9) | 5.9 (2.7-12.9) |
| 30-44 | 2.8 (1.7-4.7) | 2.4 (1.1-4.9) | 3.1 (1.6-6.2) |
| 45-64 | 1.8 (1.1-2.9) | 1.6 (0.8-3.3) | 1.9 (1.0-3.5) |
| 65-79 | ref. | ref. | ref. |
| **Residential area (inhabitants)** | | | |
| Big-city (100,000+) | 2.8 (2.0-4.1) | 2.7 (1.7-4.4) | 3.0 (1.9-4.8) |
| Medium-sized town | 1.3 (0.9-2.0) | 1.1 (0.6-1.9) | 1.8 (1.2-2.8) |
| (20,000 - <100,000) |  |  |  |
| Small-town | 1.3 (0.8-1.9) | 1.2 (0.7-2.0) | 1.3 (0.7-2.3) |
| (5,000 - <20,000) |  |  |  |
| Rural (< 5,000) | ref. | ref. | ref. |
| **Marital status** | | | |
| Single | 1.3 (0.9-1.8) | 1.4 (0.9-2.3) | 1.1 (0.6-1.8) |
| Divorced/widowed | 1.0 (0.6-1.6) | 1.3 (0.7-2.4) | 0.8 (0.4-1.4) |
| Married | ref. | ref. | ref. |
| **Socioeconomic status** | | | |
| Low | 1.5 (1.1-2.0) | 1.2 (0.8-1.9) | 2.0 (1.3-3.1) |
| Medium | ref. | ref. | ref. |
| High | 1.5 (1.1-2.0) | 1.1 (0.7-1.6) | 2.3 (1.6-3.3) |
| **Long working hours (≥50h/week)** | | | |
| Long working hours | 1.1 (0.8-1.6) | 1.1 (0.8-1.6) | 1.5 (0.7-3.4) |
| Non-working/65+ years | 1.0 (0.7-1.3) | 1.1 (0.7-1.7) | 1.1 (0.7-1.6) |
| No long working hours | ref. | ref. | ref. |
| **General state of health** | | | |
| Very good/good | 1.2 (0.8-1.8) | 1.4 (0.8-2.4) | 1.0 (0.6-1.8) |
| Average/poor/very poor | ref. | ref. | ref. |
| **Chronic disease** | | | |
| Any chronic disease | 0.4 (0.3-0.6) | 0.4 (0.3-0.7) | 0.5 (0.3-0.8) |
| No chronic disease | ref. | ref. | ref. |
| **Health insurance** | | | |
| Private | 2.0 (1.4-2.8) | 2.1 (1.4-3.2) | 1.8 (0.9-3.4) |
| Others ^d^ | 2.1 (1.4-3.1) | 2.4 (1.5-3.8) | 1.5 (0.8-2.8) |
| Statutory | ref. | ref. | ref. |

^a^Adjusted odds ratios estimated from logistic regression for the total study population (n=7.176). Nagelkerke’s R^2^: 0.14, 91% correctly classified.

^b^Adjusted odds ratios estimated from logistic regression restricted to male participants (n=3.400). Nagelkerke’s R^2^: 0.14, 89% correctly classified.

^c^Adjusted odds ratios estimated from logistic regression restricted to female participants (n=3.776). Nagelkerke’s R^2^: 0.15, 93% correctly classified.

^d^ “Others” include no insurance at all, direct payer, a foreign health insurance or any other kind of reimbursement
